# Class II correction by maxillary en masse distalization using a completely customized lingual appliance and a novel mini-screw anchorage concept – preliminary results

**DOI:** 10.1186/s13005-021-00273-3

**Published:** 2021-06-29

**Authors:** Frauke Beyling, Elisabeth Klang, Eva Niehoff, Rainer Schwestka-Polly, Hans-Joachim Helms, Dirk Wiechmann

**Affiliations:** 1 Private Practice, Kieferorthopädische Fachpraxis, Lindenstraße 44, 49152 Bad Essen, Germany; 2 Department of Orthodontics, Hannover Medical School (MHH), Hannover, Germany; 3 Department of Medical Statistics, University Medical Center Göttingen (UMG), Göttingen, Germany

**Keywords:** Mini-screws, TADs, Anchorage, Lingual orthodontics, Lingual appliances, Distalization, Class II correction

## Abstract

**Background:**

The aim of the study was to evaluate the efficacy of a novel en masse distalization method in the maxillary arch in combination with a completely customized lingual appliance (CCLA; WIN, DW Lingual Systems, Germany). Therefore, we tested the null-hypothesis of a significant deviation from an Angle-Class I canine relationship and a normal overjet defined by an individual target set-up after dentoalveolar compensation in Angle Class II subjects.

**Methods:**

This retrospective study included 23 patients, (m/f 3/20, mean age 29.6 years (min/max, 13.6/50.9 years)), with inclusion criteria of an Angle Class II occlusion of more than half a cusp prior to en masse distalization and treatment completed consecutively with a CCLA in combination with a mini-screw (MS) anchorage for uni- or bilateral maxillary distalization (12 bilateral situations, totalling 35). Plaster casts taken prior to (T0) and following CCLA treatment (T3) were compared with the treatment plan / set-up (TxP, with a Class I canine relationship and a normal overjet as the treatment objective). MSs were placed following levelling and aligning (T1) and removed at the end of en masse distalization at T2. Statistical analysis was carried out using Schuirmann’s TOST [*two one-sided tests*] equivalence test, based on a one-sample t-test with α = 0.025 on each side (total α = 0.05).

**Results:**

Ninety-seven percent of planned correction of the canine relationship was achieved (mean 3.6 of 3.7 mm) and also 97 % of the planned overjet correction (mean 3.1 of 3.2 mm), with a statistically significant equivalence (*p* < 0.0001) for canine relationship and overjet between the individual treatment plan (set-up) and the final outcome. Adverse effects were limited to the loss of *n* = 2 of 35 mini-screws. However, in each instance, the treatment was completed, as scheduled, without replacing them. Accordingly, the null-hypothesis was rejected.

**Conclusions:**

The technique presented allows for a predictable correction of an Angle-Class II malocclusion via dentoalveolar compensation with maxillary en masse distalization.

## Introduction

Distalization in the upper jaw for correction of a class II malocclusion by dentoalveolar compensation has had a long history in orthodontic mechano-therapy [1–3]. The introduction of skeletal anchorage with the aid of adapted dental implants or mini-screws (MSs), in particular, has contributed to the more widespread use of this method, since maxillary retraction or distalization can be performed with it, for example, largely independently of patient compliance, i.e., in a more controlled way [4–9]. However, if Class II malocclusion is intended to be corrected primarily by movements of the maxillary teeth, not only a reliable distalizing mechanic is required, but also torque control, to the fullest extent possible, in the area of the upper incisors and canines, during the entire stage of maxillary retraction. Moreover, perfect levelling of the mandibular arch is a sine qua non condition, as failure to achieve it will result in an outcome with class I canine relationship being impossible, just as in the case of Class II compensation with the help of maxillary premolar extraction. While the efficacy of skeletally anchored appliances for the distalization of the lateral upper teeth has been examined in some studies employing a retrospective approach based on lateral x-rays and digital cast superimpositions no investigations have been undertaken to assess such a treatment approach focusing on the post-treatment occlusion as one indicator of treatment success, along with the maxillary distalization primarily in the area of the first molars. For clinicians, these kind of considerations are of particular interest, since it is of little value to them if molar distalization succeeds but problems occur during the subsequent retraction of the upper anterior teeth, thereby preventing an outcome with a class I canine relationship.

The distalization of the entire maxillary dentition can also be performed using lingual appliances combined with skeletal anchorage, as has been described in a small number of case reports [7, 10–12]. In addition to aesthetic advantages, lingual treatment in adolescents offers the advantage of a reduced risk of enamel decalcification [13]. Also, because of the biomechanical characteristics of lingual orthodontics, torque control during upper anterior retraction is of particular importance in this situation [14, 15].

### Study objective

The aim of the study was to evaluate the efficacy of a novel en masse distalization method in the maxillary arch, in combination with a completely customized lingual appliance (CCLA). Therefore, we tested the null hypothesis that the planned and achieved corrections of canine relationship and overjet in Class II patients would be statistically non-equivalent.

## Subjects and method

### Inclusion and exclusion criteria

Subjects were consecutively recruited from a pool of patients treated with a CCLA (WIN, DW Lingual Systems GmbH, Bad Essen, Germany) in a specialized orthodontic clinic (Prof. Wiechmann, Dr. Beyling, and Colleagues, Bad Essen, Germany) which was de-bonded in the time period from January 2016 to June 2019, based on the following inclusion criteria:

- Angle Class II malocclusion of half of a cusp or more after levelling and aligning measured in the canine-premolar area.
- Treatment completed with a CCLA in combination with interradicular MS anchorage for maxillary uni- or bilateral en masse distalization.

To minimize the risk of bias, no patient was excluded from this retrospective analysis for any reason other than the defined inclusion criteria, i.e., excluded due to missed appointments, lack of compliance or missing records, as is occasionally seen in sample compositions of retrospective studies.

Of the 1393 subjects screened for potential eligibility, this study included a total of 23 patients (m/f 3/20, mean age 29.6 years, min - max 13.6–50.9 years, SD 11.5), 12 of whom had bilateral and 11 had a unilateral MS anchorage, providing a total of 35 individual MS distalization sites (Table 1).

**Table 1:** Overview of age, sex and MS insertion sites at T0

Age (mean ± SD, [min - max])	29.6 ± 11.5 [13.6 - 50.9]
Sex, n	23
male	3 (13.1%)
female	20 (86.9%)
Position MS sites, n	35
right	17 (48.6%)
left	18 (51.4%)
one side	11 (47.8%)
both sides	12 (52.2%)

### Novel mechanics for maxillary en masse distalization

The distalization concept combined to a lingual appliance retrospectively examined by this study follows suggestions by Park et al. (2004), who used one palatal mini-screw per side inserted in the interradicular region [7]. As opposed to other approaches, in which a palatal superstructure anchored to mini-screws or implants is intended to distalize the first and second molars, the objective of the approach examined in this paper is a complete en masse distalization of the entire upper arch in one step.

**Fig. 1:**
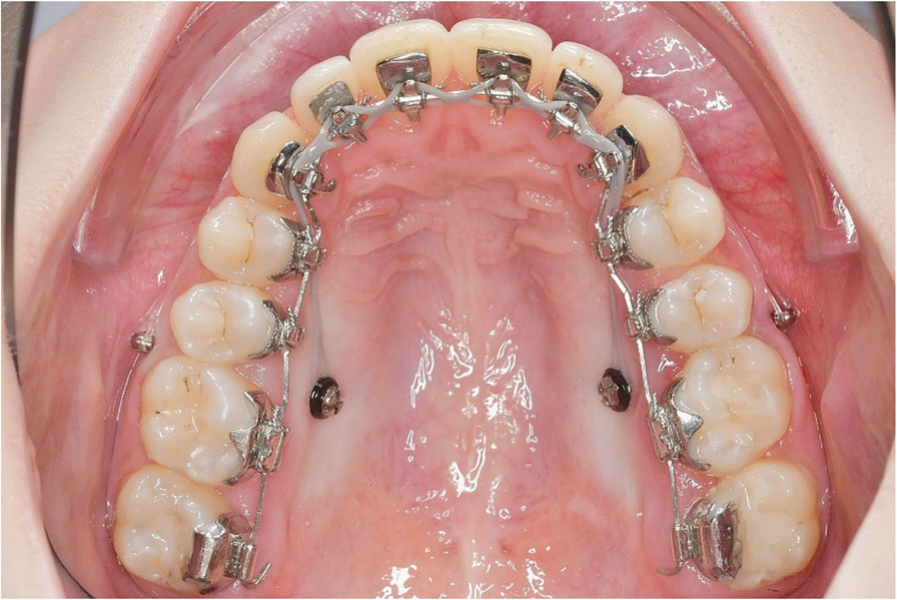
Completely customized lingual appliance (CCLA) combined with a novel mini-screw anchorage concept for maxillary en masse distalization. The 0.016’’ x 0.024’’ stainless steel archwire has an extra-torque of 13° from canine to canine and 2 cm expansion in the region of the first molars

In contrast to the method presented by Park et al., however, 2 mini-screws per side were inserted in the distalization concept evaluated in this study. Figure 1 shows the mechanics used in combination with a CCLA for en masse distalization in the upper arch. The entire maxillary dentition is moved in a posterior direction using two mini-screws per side to which elastic chains (Morita Energy Chain, Rocky Mountain Orthodontics, Denver, CO, USA) were attached. The traction force per screw should not exceed 1.5–2 N, as an excessive tipping moment may result in loose or lost screws [16, 17]. The en masse distalization is effected with a 0.016” x 0.024” stainless steel archwire (ribbon wise) with a 13 or 21 deg. extra-torque from canine to canine. Considering the limited interradicular space, the vestibular screws (Abso Anchor SH 1312–10, Feanro Ltd., Zurich, Switzerland) should be removed 3–5 months after the start of retraction, in order not to interfere with continuing distalization. The palatal screw (Dual Top S16-G2-010 N, Promedia Medizintechnik, Siegen, Germany) is inserted close to the palatal molar root, i.e. not on the midline between adjacent teeth, but, instead, in a slightly more distal location. The palatal screws are inserted perpendicularly relative to the alveolar process; the vestibular screws exhibit a clear cranial orientation, as is readily visible (Fig. 4c and d). In this way, the tip of the screws ended up in a bony area with a longer interradicular distance [18].

### Assessment of canine relationship and overjet

Plaster casts taken prior to bonding (T0) and following de-bonding of the CCLA (T3) were assessed and compared to the treatment plan represented by an individual set-up (TxP). Wax bites taken in the subject’s habitual intercuspation were used to position upper and lower plaster casts correctly without the help of a dental articulator [19]. The plaster target set-up models were evaluated mounted in the articulator. A baseline value of 0 mm was assigned in cases of an Angle Class I canine relationship (summit of upper canine’s crown corresponding with approximal contact of lower canine/first premolar). Deviations towards Angle Class II were, by definition, assigned positive values. For better visualization of Class II and overjet correction, high-resolution, digital, intraoral photographs (Camera D200, with Nikkor 105 mm, Nikon, Tokyo, Japan) were taken in habitual intercuspation after levelling and aligning (T1) and at the end of distalization (T2). They were taken directly (perpendicular to the canine’s labial surface, to avoid any potential errors caused by distortion), using cheek retractors (NOLA, Chicago, IL, USA), without using mirrors. In order to obtain the true dimensions on the digital photographs, a calibration technique which had been previously proposed was employed [20].

### Method error analysis

Repeated measurements on plaster models and intraoral photographs of ten randomly selected patients with MSs on the right-hand side were performed by the same examiner. In each patient, the canine relationship at T0 and T1, as well as the overjet at T0 and T1, were assessed. Dahlberg’s method error ranged from 0.11 to 0.23 mm for all four linear measurements, never exceeding 1 mm for any patient [21].

### Statistical data analysis

To assess the quality of the CCLA treatment, the measurement data for the canine relationship and overjet were analysed descriptively, using mean and standard deviation (SD), as well as minimum and maximum values (min - max) at the various time points under consideration. The primary endpoints were the canine relationship and overjet.

To evaluate whether the results of the primary endpoints after treatment (T3) did not differ substantially from the set-up (TxP), a test for equivalence, based on the difference (TxP-T3), was used to assess if the mean difference and corresponding 95 % CI lay within the pre-specified tolerance interval of ± δ=± 0.5 mm around the optimum of no difference (difference = 0). The analysis carried out using Schuirmann’s TOST (*two one sided tests)* equivalence test, based on a one-sample t-test with α = 0.025 on each side (total α = 0.05). All statistical analyses were carried out using the statistical software SAS version 9.4 (SAS Institute, Cary, NC, USA).

### Retrospective power calculation

With the available sample size of 35 MS-distalization sites in 23 patients, a retrospective power calculation was performed for the canine relationship and overjet, in order to assess the difference in treatment results (T3) vs. set-up (TxP) (difference = TxP - T3).

For the results at T3 to be comparable to the planned set-up TxP, a test for equivalence based on the difference was used to assess whether the difference was within a tolerance interval ± δ around the optimum (difference = 0). It was assumed that a deviation from the optimum up to δ = 0.5 mm in either direction would be clinically acceptable. For both retrospective power calculations, the error was set to α = 0.025 on each side (over- and under-correction) and the power to 80 %.

For the canine relationship (35 MS-distalization sites): With an assumed standard deviation of SD = 0.5 and an expected difference (TxP-T3) of less than 0.25 mm, the statistical equivalence can be shown with a power of 80 %.

For the overjet (23 patients): With an assumed standard deviation of SD = 0.4 and an expected difference (TxP-T3) of less than 0.25 mm, the statistical equivalence can be shown with a power of 80 %.

## Results

MS-supported en masse distalization took on average 10.5 ± 4.5 months (min. 5.2, max. 19.8), with a mean correction of the class II canine relationship of 0.43 mm per month from T1 to T2. For the earlier cases (MS placed in 2014 and 2015, 14 MS-distalization sites), the mean duration of en masse distalization was 13.5 ± 4.2 months (min. 6.0, max. 19.8), with an average correction of the class II canine relationship of 0.33 mm per month. And for the later cases (MS placed 2016 and later, 21 MS-distalization sites), the duration of en masse distalization was substantially reduced, to an average of 8.5 ± 3.6 months (min. 5.2, max. 17.5), with an average correction of the class II canine relationship of 0.49 mm per month.

Adverse effects in relation to the MS anchorage were limited to the loss of n = 2 of 35 MSs in 2 different lateral segments. As the second screw in each particular segment was still serviceable on both occasions, the treatment was completed as scheduled without replacing the MSs.

### Correction of canine relationship

**Fig. 2:**
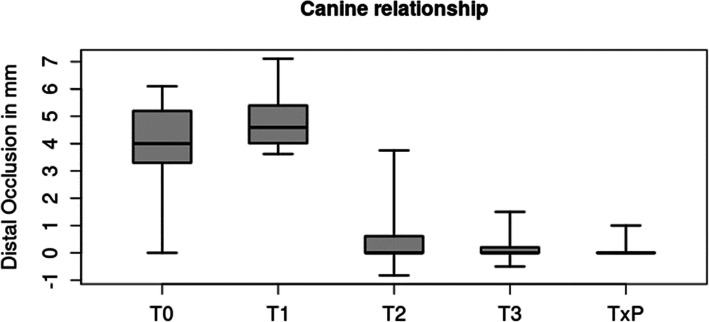
Boxplot of canine relationship over the different time points (T0, T1, T2, T3) and treatment plan (TxP) defined by an individual set-up. Showing Median, interquartile range (IQR) and Min-Max

The initial mean canine relationship of 3.8 ± 1.9 mm worsened to 4.8 ± 1.0 mm during levelling and aligning due to the clockwise rotation of the mandible after inserting the lingual appliance. During MS-supported en masse distalization, it was corrected to a mean of 0.5 ± 0.9 mm and further improved, in finishing, to a mean of 0.2 ± 0.5 mm. Compared to the individual treatment plan TxP (mean: 0.1 ± 0.3 mm), 97 % of the planned bite correction could be achieved T0-T3 (Tables 2 and 3a, Fig. 2).

**Table 2:** Canine relationship and overjet at the different time points and on the individual target set-up (TxP)

	Canine relationship [mm] *n* = 35		Overjet [mm] *n* = 23	
	mean ± SD	[min - max]	mean ± SD	[min - max]
T 0	3.8 ± 1.9	[ 0.0 - 6.1]	5.4 ± 1.9	[2.5 - 10.0]
T 1	4.8 ± 1.0	[ 3.7 - 7.1]	5.5 ± 1.6	[2.0 - 8.3]
T 2	0.5 ± 0.9	[ -0.8 - 3.8]	2.4 ± 0.7	[1.2 - 3.9]
T 3	0.2 ± 0.4	[ -0.5 - 1.5]	2.3 ± 0.3	[ 2.0 - 3.0]
T x P	0.1 ± 0.3	[ 0.0 - 1.0]	2.2 ± 0.5	[ 1.0 - 3.0]

**Table 3:** a/b: Treatment effect on canine relationship (a) and overjet (b) in different time intervals

a) Canine relationship					
	T3 – T0	TxP – T0	TxP –T3	T3 – T1	T3 – T2
n	35	35	35	35	35
mean ± SD	-3.6 ± 1.8	-3.7± 1.8	-0.1 ± 0.4	-4.6 ± 1.0	-0.3 ± 1.0
[min – max]	[-6.1 – 0.4]	[-6.1 – 0]	[-1.5 – 1.0]	[-6.8 – -2.9]	[-3.8 – 2.1]
b) Overjet					
	T3 – T0	TxP – T0	TxP –T3	T3 – T1	T3 – T2
n	23	23	23	23	23
mean ± SD	-3.1 ± 1.7	-3.2 ± 1.8	-0.1 ± 0.4	-3.2 ± 1.5	-0.1 ± 0.6
[min – max]	[-7.5 – -0.5]	[-7.0 – -0.5]	[-1.0 – 1.0]	[-6.3 – 0.0]	[-1.4 – 1.2]

There was a statistically significant equivalence (*p* < 0.0001) for canine relationship between the individual treatment plan (set-up) and the final outcome (mean difference=-0.16 mm, SD = 0.5, 95 % CI -0.25, 0.04). 97 % of the planned correction was achieved (3.7 mm planned, 3.6 mm achieved).

### Correction of overjet

**Fig. 3:**
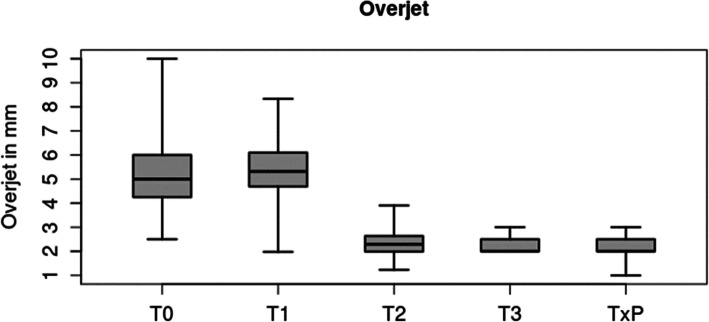
Boxplot of overjet over the different time points (T0, T1, T2, T3) and treatment plan defined by an individual set-up. Showing Median, Interquartile Range (IQR) and Min-Max

The initial mean overjet of 5.4 ± 1.9 mm remained relatively stable in levelling and aligning (5.5 ± 1.6 mm at T1). During MS-supported en masse distalization, the overjet was reduced to a mean of 2.4 ± 0.7 mm and further improved slightly during finishing to a mean of 2.3 ± 0.3 mm. Compared to the individual treatment plan TxP (2.2 ± 0.5 mm), 97 % of the planned overjet correction could be achieved T0-T3 (Tables 2 and 3b, Fig. 3).

There was a statistical equivalence (*p* < 0.0001) for overjet between the individual treatment plan (set-up) and the final outcome (mean difference=-0.06 mm, SD = 0.43, 95 % CI -0.253, 0.123). 96 % of the planned correction was achieved (3.2 mm planned; 3.1 mm achieved).

The null hypothesis of a significant deviation from the planned canine relationship and overjet (TxP) after using a CCLA in combination with a novel maxillary MS anchorage in Angle Class II subjects was rejected.

## Discussion

This paper is the first investigation of the reliability of en masse distalization in the upper arch combined with a lingual appliance. Based on a lingual case report, Park et al. (2004) described the use of palatally inserted interradicular MSs for maxillary en masse distalization combined with a lingual appliance [7]. In combination with vestibular appliances, interradicular MSs were inserted on the buccal side. Compared to this, previous studies have only explained the mechanics with the help of case reports [7, 18, 22, 23], whilst later studies assessed the efficacy of such mechanics based on molar distalization in the cephalogram [8, 24–26]. Up until now, an evaluation of the bite correction achieved, as well as of the post-treatment canine relationship, has never been carried out. This is even more surprising, since occlusal and dental parameters evaluated on plaster casts constitute a common standard for evaluating the quality of treatment results. Therefore, the treatment outcome was not evaluated in this study by an uni-maxillary assessment based on the position of the upper molar in the cephalogram, but by a bi-maxillary consideration of the entire occlusion, which was then compared to the treatment plan (TxP, individual target set-up). Therefore, this study was explorative in nature, hence its retrospective design. However, all patients who were de-bonded within the time frame of forty-two months were screened for eligibility prior to commencement of any assessments. In order to evaluate the predictability of the concept in this consecutive sample, no patient who met the inclusion criteria was excluded for any secondary reason (lack of compliance, missing records, inadequate oral hygiene, or similar reasons).

The main objective of this study was to analyse the predictability of achieving the desired Class I occlusal relationship and overjet as defined by an individual target set-up. A Class I occlusal relationship defines the gold standard when assessing the outcome of an orthodontic treatment, as described by various scores [27–29]. Therefore, we did not use radiographic landmarks to evaluate molar distalization, as has frequently been proposed by various authors [24, 30–43]. The measurement method, which was introduced previously, is relatively simple and can be applied to each lateral segment, separately, which is of major importance particularly in asymmetric Class II cases [7, 44]. Dahlberg’s method error assessment showed a high reproducibility of the measurements.

Despite the growing popularity of implants placed in the anterior palate, the alveolar bone still seems to be a common region for MS insertion. If the screw type is well adapted to the insertion site and if the proposed guidelines for placement and loading are respected, implant loss before schedule is a rare complication. In our study, only 2 out of 70 MSs were lost before schedule, indicating a survival rate of over 95 %. This is in agreement with the findings of Berens et al., who reported a failure rate of less than 10 % for palatal and buccal maxillary interdadicular locations when choosing a screw geometry that is well adapted to the space available [45]. A recent systematic review reported a similar loss rate for the interradicular insertion sites in the maxilla [46]. Moreover, these authors also found a slightly lower average loss rate for implants placed in the midpalatal and paramedian region (0–5 %). As described by Park et al., the buccal interradicular screw should be inserted at a 30 deg. angle with respect to the long axis of adjacent teeth, with its head about 3–4 mm above the gingival border [18]. Placed this way, a 3.5 mm distalization should be possible [7, 47]. The palatal screw should be placed as close as possible to the palatal root of the first molar, perpendicular to the alveolar process, with its head about 3–4 mm above the gingival border. Positioned in this way, adverse effects, such as root damage, are unlikely to occur and would be mainly without clinically relevant consequences [48]. In order to counteract the higher tip moment caused by the longer lever arm, due to the thicker gingiva, the palatal screw should be thicker (1.6 mm) than the buccal one, as described by Berens et al. [44]. Each screw can be loaded with 1.5–2 N without reaching the critical tip moment that would put the screw at risk [16, 17]. The use of four screws for en masse distalization generating a fully friction-free distalizing force of 3–4 N for each lateral segment is in line with the findings of two systematic reviews and seems to be reasonable, as not only the upper first molars should be moved distally, but the entire maxillary dentition [49, 50]. Although one screw was lost in two patients in this study, an additional surgical procedure to insert a new screw in a different location could be avoided, as the second, remaining screw was still serviceable.

### Indications

**Fig. 4:**
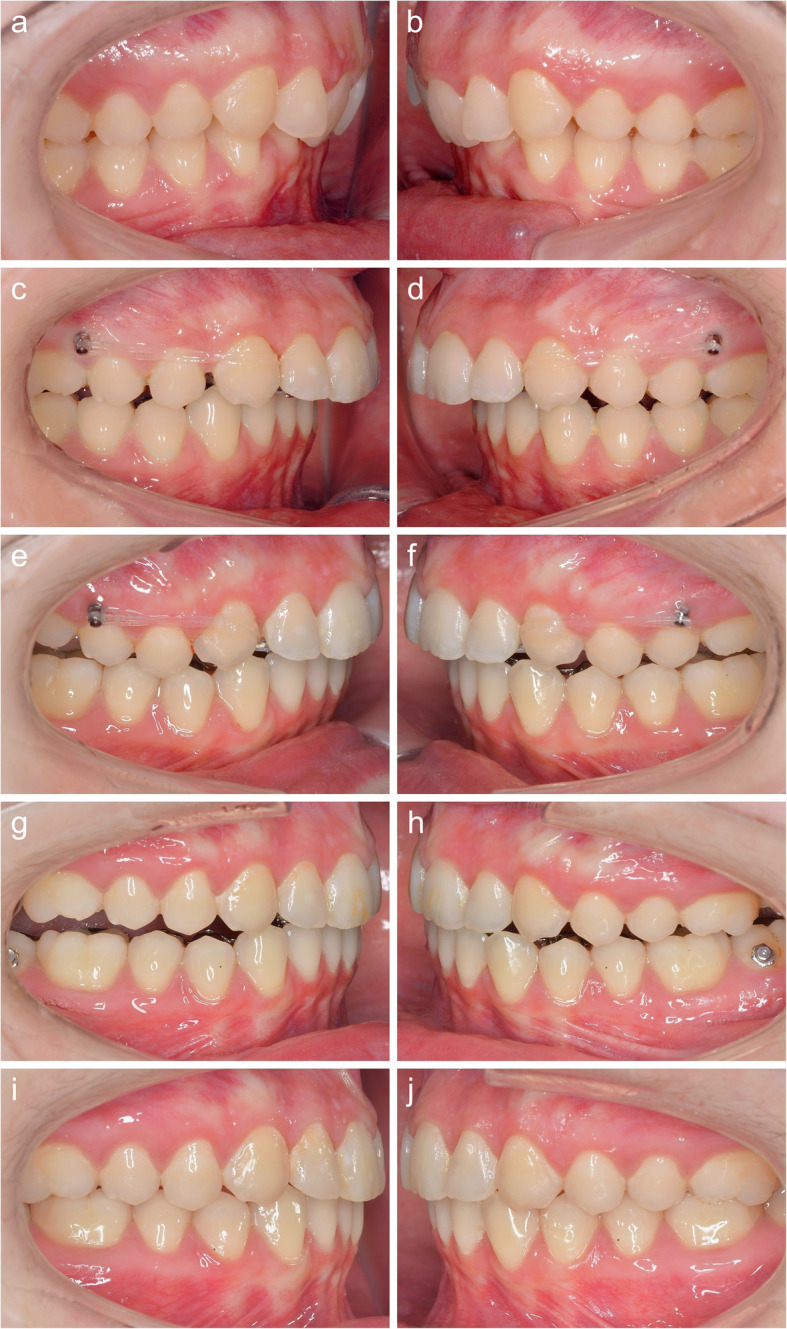
The initial class II malocclusion (**a,b**, T0) is worsening to more than half a unit during levelling and aligning (**c,d**). At the beginning of en masse distalization (T1), the lower curve of spee is levelled and the inclination of the upper incisors has improved (**c,d**). The buccal MSs are inserted with a 30 degree angle compared to the adjacent teeth. A few months later, the overjet is reduced and now the buccal screws have to be removed (**e,f**). Further bite correction achieved with the help of the palatal screws only, which now are removed (T2). Note the intrusion in the upper lateral segments (**g,h**). Final result (T3) after maxillary en masse distalization with upper torque control (**i,j**). A bilateral class I canine relationship and a normal overjet could be achieved

**Fig. 5:**
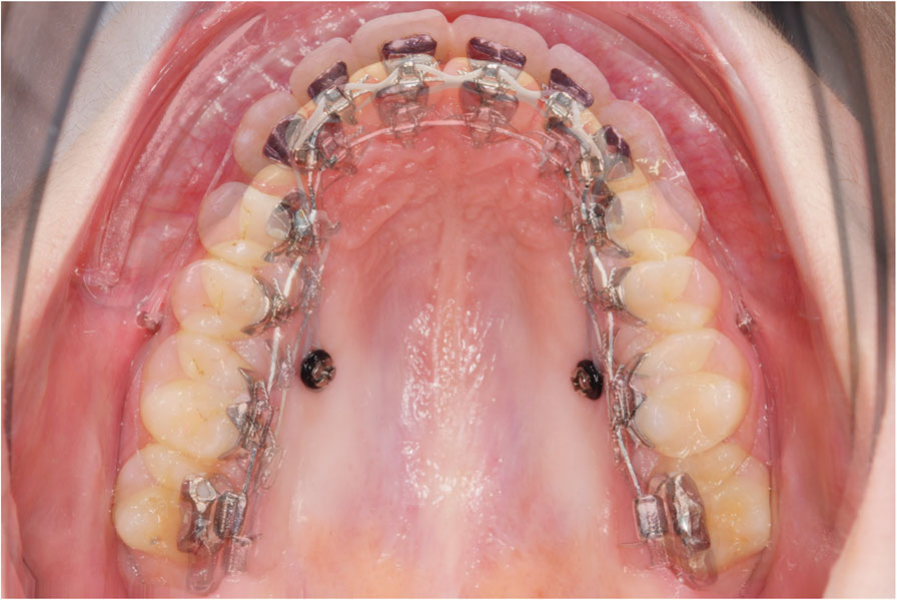
Superposition of the maxillary dentition at T1 and T2 on the palatal MSs (for illustrative purposes only, not used for measurements!). The distance between the two archwires in the anterior segment is indicating the amount of maxillary en masse distalization

In the early patients of our cohort, the novel distalization concept was selected as an alternative to the initial treatment plan (intermaxillary elastics) due to poor compliance. The fact was that those patients, in particular, for aesthetic reasons, in many cases rejected a visible alternative to Class II correction, such as a Herbst appliance or flexible bite jumpers. Having established viability, the novel distalization concept was used in later patients from our cohort as an alternative to maxillary premolar extraction, too (Fig. 4). In all of these cases, Class II was meant not to be corrected by a substantial mesial movement in the mandible, but mostly by retraction in the maxilla (Fig. 5). The concept also enables sizeable unilateral maxillary distalization, turning it into an option of choice for patients with an asymmetric occlusion and upper midline discrepancy. This holds, in particular in cases, in which unilateral premolar extraction is out of the question.

### Preconditions for successful en masse distalization

In contrast to previous studies, in which retrospective analysis mostly focused, in isolation, on the efficacy of molar distalization when vestibular appliances were used, this study assessed the full duration of Class II correction, including of anterior retraction and achievement of a class I canine relationship. In this respect, along with the distalization proper using MSs, the levelling of the mandibular curve of Spee and control of the torque in the maxillary anterior region during retraction in particular are of great importance. To achieve the outcome represented by the treatment plan (TxP), it is essential to address all three treatment tasks: levelling in the mandible and distalization with anterior torque control in the maxilla. With a CCLA, the orientation of the archwire plane is ribbon wise, thereby favouring mandibular levelling. The effective torque control that can be achieved with the lingual appliances used has been demonstrated by a number of in vitro and in vivo studies [14, 51–55]. But, in particular, in instances in which innovative mechanics are used, the clinician will always be subject to some kind of learning curve, in order to make full use of the performance potential of the novel development. In this study, the learning curve can be recognized from the substantially shorter average duration of the distalization stage (T1-T2) in later treatments compared to earlier patients (8.5 months with a speed of 0.49 mm per month compared to 13.5 months with a speed of 0.33 mm per month). This was mainly due to the increase in the anterior extra-torque in the 0.016” x 0.024” stainless steel archwire from 13 deg. to 21 deg. for improved torqueing during retraction and also to a 2 cm expansion in the area of the first molar achieved in the same archwire. This expansion proved very valuable, as the anterior torque applied with a view to torqueing during retraction results in intrusion in the lateral segments, which leads to compression for the dental arch when lingual appliances are used. The maxillary posterior intrusion as such has a favourable effect on mandibular repositioning, as it prevents largely an unwanted clockwise rotation of the mandibular jaw.

### Amount and pattern of bite correction

The average bite correction achieved from T1 to T3, a mean 4.6 ± 1.1 mm (min/max 2.9/6.8), cannot be compared immediately, due to the differences in method, to the outcomes reported by Yamada et al. and Sang et al., who found maxillary molar distalization in the cephalogram of, respectively, a mean 2.8 mm and 2.0 mm for a distalization using buccally inserted interradicular MSs in combination with labial appliances [26, 56]. Utilising a comparable method, Bechthold et al. described distalization with a mean up to 2.9 mm [24]. Longer average lengths of distalization of maxillary molars, even more than 6 mm in the area of the dental crown, have been described in cases of insertion of the MSs in the anterior palate, though some studies have found considerable distal tipping of the upper first molars [57]. Using a rigid superstructure, Nienkämper et al. reported average distalization in the area of the centre of resistance of the upper first molar of 3.6 ± 1.9 mm (min/max 1.2–8.5 mm) [40]. Thanks to the control achieved, distal tipping of the upper first molars could be limited to 1.5 deg. on average in this case [40, 58]. However, studies on distalization appliances anchored to the palate can be compared to this paper only to a very limited extent, since the analyses performed for them mostly focus on molar distalization in the maxilla only. Essential treatment tasks, including mandibular levelling and maxillary retraction from 5 to 5 ensuring anterior torque control, remain to be done at that point and in part pose a considerable challenge in terms of quality of posterior anchorage [31–36, 39–43].

Looking at the canine relationship at various time points, a clearly worse situation at the end of levelling and aligning, T1-T2, is immediately noticeable. This is a characteristic of lingual treatment, in which, in particular in cases of deep bite, the premature tooth contacting the appliance results in a clockwise rotation of the mandible and displacement towards caudal and towards posterior. Moreover, to establish the optimum inclination of the upper anterior teeth by palatal torqueing on the root, force directed towards posterior is required, which also results in a mesial effect on the upper lateral teeth [59]. Both effects combined will lead to a canine relationship worsened by almost 1 mm, on average (Fig. 4c and d). These movements do not exercise any considerable influence on the mean overjet, as the referred-to effects will be compensated, almost completely, by mandibular proclination, in order to resolve crowding and reclination of upper anterior teeth in Class II/1 patients. The slight improvement, on average, at T2-T3 (canine relationship: 0.2 mm, overbite 0,1 mm) can be explained by the occlusion optimization during finishing. The grinding of all premature contacts on the appliance in the area of the occlusal pads on the second molars and the upper anterior teeth will lead to a slight, counter-clockwise rotation of the mandible which, in turn, improves the canine relationship and the overjet minimally. Remaining differences with respect to the individual treatment planning, as represented by the target set-up, amounting to a mean 0.16 mm/0.06 mm (canine relationship/overjet) and a maximum under-correction of 1.0 mm, are clinically acceptable, which emphasises the performance potential of fixed orthodontic appliances for the correction of Angle Class II malocclusions in adult patients, compared to approaches employing removable appliances [60].

## Conclusions

- The technique presented allows for successful correction of Angle Class II malocclusions by dentoalveolar compensation, mainly with maxillary tooth movements.
- Preconditions for successful Class II correction, such as lower levelling and upper incisor torqueing, can be accomplished using fixed lingual appliances.
- Maxillary en masse distalization with the presented novel concept can be a reliable alternative for dentoalveolar Class II correction when major mesialization of the mandibular dentition is not desired.

## Data Availability

The datasets used and/or analyzed during the current study are available from the corresponding author on reasonable request.
